# Predator-Specific Effects on Incubation Behaviour and Offspring Growth in Great Tits

**DOI:** 10.1371/journal.pone.0121088

**Published:** 2015-04-01

**Authors:** Alessandra Basso, Heinz Richner

**Affiliations:** Institute of Ecology and Evolution, University of Bern, Baltzerstrasse 6, 3012, Bern, Switzerland; Hungarian Academy of Sciences, HUNGARY

## Abstract

In birds, different types of predators may target adults or offspring differentially and at different times of the reproductive cycle. Hence they may also differentially influence incubation behaviour and thus embryonic development and offspring phenotype. This is poorly understood, and we therefore performed a study to assess the effects of the presence of either a nest predator or a predator targeting adults and offspring after fledging on female incubation behaviour in great tits (*Parus major*), and the subsequent effects on offspring morphological traits. We manipulated perceived predation risk during incubation using taxidermic models of two predators: the short-tailed weasel posing a risk to incubating females and nestlings, and the sparrowhawk posing a risk to adults and offspring after fledging. To disentangle treatment effects induced during incubation from potential carry-over effects of parental behaviour after hatching, we cross-fostered whole broods from manipulated nests with broods from unmanipulated nests. Both predator treatments lead to a reduced on- and off-bout frequency, to a slower decline in on-bout temperature as incubation advanced and showed a negative effect on nestling body mass gain. At the current state of knowledge on predator-induced variation in incubation patterns alternative hypotheses are feasible, and the findings of this study will be useful for guiding future research.

## Introduction

Birds can assess predation risk and adjust behaviours adaptively [1], for example by reducing investment in current reproduction [2,3]. To reduce the risk of predation, birds may minimize activity around the nest [4], increase vigilance [5], alter incubation behaviours [6,7], feeding habits [8], or patterns of parental care. The involved trade-offs between investment in current reproduction versus self-maintenance should depend on variation in predation risk [1,9] and type of predator [9]. Predators may differ in imposed risk levels, hunting strategies or target for example during reproduction where either parents or offspring may be at higher risk. Prey responses should therefore be adjusted to the type of predator encountered [10,11]. For example, male pied flycatchers show different behavioural and hormonal responses when exposed to the great spotted woodpecker or a weasel, both common predators of this species [12], and Passerines use different alarm calls depending on predator size [13] and type [11]. During incubation females are constrained to a specific nest location, and under high predator abundance face considerable risks. As a consequence, incubation strategy is also predicted to vary as a function of predator pressure. In species where females only perform incubation and hence face a strong trade-off between investment in incubation and self-maintenance [4,14], males can assist by providing food at the nest [15], or lead females to rich feeding sites [16]. Trips from and to the nest can be minimized to avoid revealing nest location [4,17] and incubation temperature should be increased in order to reduce the number of days eggs are exposed to predators [4,18].

The effects of predation risk on incubation behaviour of birds are still poorly understood. To the best of our knowledge it has never been investigated whether the type of predator encountered may differentially alter incubation behaviour, depending on whether risk is directed at the parents and fledglings outside the nest, or to parents and their clutch/brood inside the nest. Parents may indeed be able to engage different mechanisms to cope with each enemy in a specific way [10]. Incubation behaviour can influence nestling growth and development [19,20], yet evidence for effects due to predation risk during incubation on offspring fitness is scarce.

**Fig 1:**
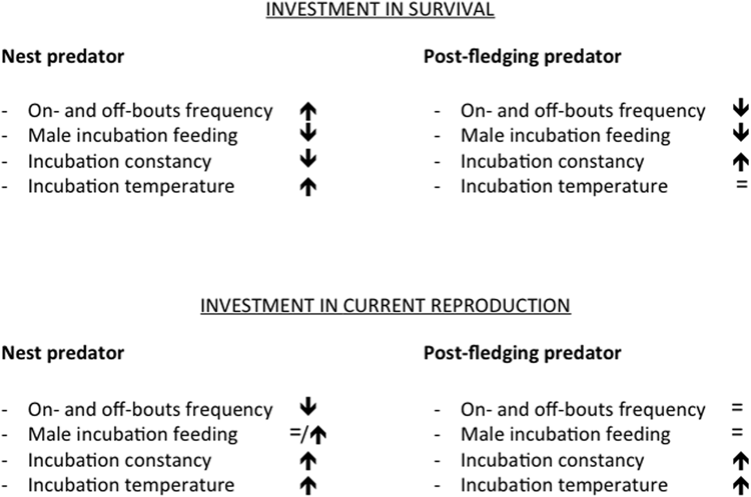
Summary of the predictions for the two maternal predator treatments.

In this study, we used a wild great tit (*Parus major*) population breeding in artificial nest boxes to test whether female incubation behaviour is differentially affected by the presence of either a nest predator (the short-tailed weasel (*Mustela erminea*)) or of a predator targeting adults during incubation and offspring after fledging (the sparrowhawk (*Accipiter nisus*)). Predictions for the direction and magnitude of indirect effects of predation on prey are not straightforward, given that they are contingent on state-dependent life-history decisions [21] (Fig. 1). On the one hand, parents may invest more in current reproduction at the expense of self-maintenance. Under the threat of a nest predator, females are then expected to minimize activity at the nest (i.e. longer and fewer on- and off-bouts) to reduce visual cues that might reveal the position of the nest, while with a post-fledging predator, on- and off-bout frequency and duration should not be altered. On the other hand, if self-maintenance and thus future reproduction are prioritized, a nest predator may induce females to leave the risky environment of the nest more often, while a post-fledging predator that is also a threat to the female while foraging may prompt females to reduce off-bout (and consequently on-bout) duration and frequency. The frequency and duration of on- and off-bouts are also expected to negatively correlate with male incubation feeding. Incubation constancy, i.e. the total time a female spends incubating during the day, is a measure of nest attendance and incubation investment. A nest predator may represent a direct risk for the survival of incubating females. If the costs of incubating under such high risk conditions overcome the benefits of investing in the current clutch, females may reduce nest attendance. In the case of a post-fledging predator, in contrast, the relatively safer environment within the nest may possibly lead the female to spend more time inside. Under both predation threats, investment in current reproduction should maximize nest attendance and the rate of embryonic development (e.g. higher incubation temperatures).

Incubation patterns may also affect offspring growth and condition. The few studies available report that sub-optimal incubation temperatures may lead to lower nestling body mass [19,20] and slower skeletal growth [22]. To explore the possibility of potential changes in nestling growth in response to maternal incubation behaviour, we swapped whole broods directly after hatching into foster nests where mothers had not experienced any treatment. This procedure allowed to separate the effects of increased predation risk during incubation on embryonic and nestling development per se from effects of maternal risk exposure on post-hatching parental care caused by a carry-over effect of predation risk during incubation.

## Materials and Methods

Great tit incubation behaviour comprises incubation sessions where females warm the eggs, and off-bouts where females are out of the nests and eggs cool down. Females only develop a brood patch and incubate, while males may assist by providing incubating females with food. Incubation lasts 12–14 days and fledglings leave the nest 18–21 days after hatching [23]. The study was conducted during spring 2013 in a natural population of great tits breeding in nest-boxes in the Bremgartenwald forest near Bern, Switzerland (46°57’N, 7°24’E). Nest boxes were distributed over 20 experimental plots holding 14 to 16 nest boxes each. Plots were approximately 120 m apart, corresponding to two great tit territories [24] in order to reduce the influence of treatments on neighbouring plots. Nest boxes were visited regularly during early spring to determine the start of laying, incubation and the hatching day (defined as day 0). From day 16 post-hatching onwards, nests were checked every afternoon to determine fledging date and the number of fledged nestlings.

We simulated increased predation risk in 6 plots (3 were allocated to the nest predator treatment and 3 to the post-fledge predator treatment), and 3 plots were assigned to a control treatment (see below). The remaining 11 plots were kept untreated and were used later as foster plots. We simulated the presence of predators near the nest boxes using stuffed models of two different predators: the sparrowhawk (Ps) as a post-fledging predator, and the short-tailed weasel (Pw) as a nest predator. As a control, we used stuffed models of the song thrush (*Turdus philomelos*) (C). Sparrowhawks are among the main predators of fledgling and adult great tits [25], while short-tailed weasels are important natural nest predators, active both during the night and during the day [26], able to enter nest boxes and representing a risk for incubating females, eggs and nestlings [12,27]. Song thrushes are not a threat or competitors for great tits. All species are naturally present in the area and they have been observed also during the period of this study. The treatment was performed at the nest level, but all the nests in a single plot received the same treatment in order to avoid the influence of the treatment on the neighbouring nests. To ensure a randomized assignment of our treatments and to avoid seasonality effects correlated with bird quality [28], we randomly created 3 blocks composed of 4 types of treatment groups, with 2 foster types in each block, plus 2 additional foster plots (to allow a higher number of brood exchanges). Once at least one nest reached the second day of full incubation, the whole plot was assigned to the first treatment in the first block, the second plot to the second treatment etc. In case more than one nest reached the predefined threshold on the same day, we rolled a dice to decide the order of assignment to one of the treatments. Treatments were properly randomized according to egg mass before the treatment (C: 12.384±0.852; Ps: 12.568±0.669; Pw: 12.644±0.631; *F*_2,6_ = 0.028, *P* = 0.972).

### Predator treatments

Since the simultaneous playback of the vocal responses to models increases the detectability of the models and helps to avoid habituation [29], we recorded predator-specific alarm responses in four great tits territories to stuffed models of sparrowhawks in the beginning of the breeding season. These territories were not part of the present experiment. For the weasel, we used specific alarm calls recorded the previous year using the same method. The same protocol was also used in four other nests exposed to the presence of song thrush models, where no alarm response occurred, to obtain natural background sounds.

Starting from the second day of incubation until the day of hatching, we performed simulations by placing the stuffed models 1 to 1.5 meters away from the nest for 10 minutes every second day to prevent habituation to the models [12,30]. Simultaneously the predator-specific alarm calls as described above were played from portable loudspeakers (Fox-Pro NX3 game caller; FOXPRO Inc. Wildlife Equipment, Lewistown, PA, U.S.A.) placed 2–3 meters away from the nest in a concealed position. Eight short-tailed weasel, six sparrowhawk and eight song thrush models were used, alongside with four soundtracks recorded in different nests for each treatment. Models, soundtracks and timing of simulations were changed randomly between simulations to prevent habituation. Despite individual variation in reaction to the predator displays, responses were observed in all territories and alarm calls or aggressive behaviour occurred in 72% of the simulations.

### Clutch mass and incubation behaviour

On the third day of full incubation we weighed whole clutches from treated nests to the nearest 0.1 g. We measured clutch temperature and incubation rhythm by placing data loggers (Voltcraft DL-111K; CONRAD, Electronic AG, Hirschau, Bayern, Germany) at the bottom plate of the nest box, and the connected thermocouple in the bottom centre of the nest cup at level with its lining, with the eggs aggregated around it. Data loggers were programmed to record the temperature every 20 seconds during incubation with a resolution of 0.1°C. Specifically, they detected changes in temperature after initiation or termination of each new incubation session (incubation rhythm) [31,32]. Data loggers were calibrated by testing them at two different temperatures: 5°C and 25°C prior to use and recorded over 10 minutes every 30 seconds. All data loggers used for the experiment appeared to be highly accurate (N = 50, mean±1SD: for 5°C = 5.4±0.18; for 25°C = 25.2°C±0.20). The correspondence of data from loggers with female incubation behaviour was verified by video-recordings at the nest. We placed a digital camcorder in each nest on day 3 of incubation, which also allowed us to monitor male incubation feeding (number of times the male fed the female). Filming was performed from 7:00 in the morning and continued for 6–7 hours. The roofs of the nest boxes were fitted with a dummy camera sticker at the beginning of the breeding season to get females used to the real camera placed later, and the first 30 minutes of each video were discarded from the analyses. Off-bout duration in video analyses was comparable to the one shown by the data loggers (Δ_off-bouts_ = 36.99 sec±11.15 sec; t_82_ = -0.65; *P* = 0.517). We used the software combination of Raven (version 1.4) and Rhythm 1.0 [33,34] to analyse temperature fluctuations and inferred duration and frequency of off-bouts. To detect off-bouts, we used a criterion of a minimum decrease of 2.0°C in egg temperature and a minimum duration of 4 minutes. All Rhythm outputs were visually inspected in Raven to verify potentially erroneous selections. Incubation behaviour was analysed for 7 consecutive days from day 4 to day 10 of incubation. We excluded the day when measures started (day 3 of incubation) and the day when the probe was removed (day 10) from the analyses to avoid confounding factors due to different starting times of recordings. We analysed on- and off-bout temperatures (calculated as the average temperature of on- and off- bouts on each active day), incubation constancy (as the total amount of time incubated during the day), average duration and number of on- and off- bouts and incubation duration. As we measured temperatures at the base of the nest cup, it is likely that our estimate is rather conservative and slightly lower than egg temperature. However, changes in temperature were easily detectable when females left or returned to the nest and started a new incubation session (as assessed by comparing thermal data and video footage). Ambient temperature was derived from a second temperature sensor (I-button, US), placed on the external side of one nest box located in a central position in each plot, while daily precipitation from the BAFU (Bundesamt für Umwelt, Switzerland) NABEL weather station, located 3 km from the study site. Both parameters were calculated as daily averages for each nest from the morning of the 4^th^ incubation day until the day the data loggers were removed (day 10).

We excluded from all analyses nests where the female displaced the probe from its initial position or where the nest was deserted or predated (N = 8). Data for the whole incubation period were successfully recorded and subsequently analysed for 47 nests (N = 15 for adult predator (Ps); N = 18 for nest predator (Pw); N = 14 for control (C)).

### Cross-fostering and nestling growth

On the third day after hatching (day 3), whole broods of similar size (± 1 nestling) and similar hatching date (± 1 day) from nests exposed to predator and control treatments were swapped with broods from foster nests. Nestlings were individually marked by removing tuft feathers from their heads, backs and wings. 56 pairs of nests were successfully cross-fostered (N = 23 for adult predator (Ps); N = 17 for nest predator (Pw); N = 16 for control (C)).

Before cross-fostering whole broods, nestlings of predator-exposed and control mothers were weighed to the nearest 0.1g. Body weight (to 0.1 g) and tarsus length (to 0.1 mm) were measured on day 8 and 15 post-hatch. On day 8 a small blood sample (Less than 5 μl) was collected from the nestlings’ meta-tarsal vein for molecular sexing using the primers 2917/3088 [35].

### Statistical Analyses

Linear mixed effect models (LMM) with restricted maximum likelihood estimation (REML) were used to evaluate the effects of the parental treatment on incubation rhythm and behaviour as incubation advanced. We assessed treatment effects (1) on incubation constancy and on- and off-bout temperatures separately (to assess female investment in keeping eggs warm), (2) on average on- and off-bout duration and duration of the incubation period, as well as (3) on brood size and (4) on fledging age. Using a generalized linear mixed model (GLMM; R package lme4, [36]) with Poisson error structure we investigated the effects of the predator treatments on the number of on- and off-bouts. Laying date, clutch size, average ambient temperature and average daily precipitation were included as explanatory variables in all initial models related to incubation behaviour. We included brood size as a covariate in the model of fledging age, and laying date, average ambient temperature and average daily precipitation in the model of brood size. To account for repeated observations performed on each nest, we used incubation day as the repeated measure and nest identity nested in plot as random factors in all models. We also added the two-way interaction between treatment and incubation day to assess possible differences in treatment effect as incubation advanced. In the models of on- and off-bout temperatures we included, in addition, a quadratic term for incubation day to allow for non-linearity, since it improved the model fit and proved significant. Since 48.5% of males never fed the female inside the nest during the recorded time on day 3 of incubation, independently of the treatment (Fisher’s exact test: *χ* ^2^ = 0.768, *P* = 0.681), male incubation feeding was modelled using a zero-inflated Poisson model, with treatment included as a factor. Using a GLMM with Poisson error structure, we also analysed male incubation feeding restricting the analyses to the nests where the male actually fed the female to evaluate possible differences related to the treatment.

Nestling morphological traits (i.e. mass gain and tarsus growth) were analysed using a repeated measurements approach in two LMMs, using age as the repeated measure. Age was treated as a categorical variable, with three levels for the model of mass (day 3, day 8 and day 15), and two for the model of tarsus (day 8 and day 15). To account for non-independence among siblings, we included nestling identity nested within nest of origin, and nested within plot as random factors. Our initial models included the two-way interaction between sex and treatment to account for potential differences among male and female nestlings, and brood size and hatching date as covariates. We also checked for correlations among nestling morphological traits and each incubation parameter. A GLMM with binomial errors was used to analyse fledging success (number of fledged nestlings over number of hatched nestlings), including brood size and hatching date as covariates.

For all models, we calculated standardized effect sizes by z-transforming the independent continuous variables following Nakagawa and Cuthill [37,38]. However, as we always had several predictors in the model, this method does provide a “semipartial” correlation, which will always be smaller than a partial correlation [38]. Average on- and off-bout duration was log transformed and nestling mass gain was square-root transformed to fit the assumptions of normality of the residuals and homoscedasticity. In the tables we provide log- and square-root transformed estimates. We included in all models plot of origin as random factor. Non-significant interactions (with *P* > 0.05) were removed to improve interpretability of main effects [39], while main effects, including covariates and treatments, were never eliminated from the model. To interpret significant interactions, we split the models according to treatment levels and examined the model summaries. Significance in GLMMs was tested via likelihood ratio tests of nested models [40] and the possibility of overdispersion was checked in all GLMMs. Unless otherwise mentioned, we report model standardized effect sizes and 95% confidence intervals.

Given the elevated number of statistical tests performed, as may be typical for an exploratory study with this kind of complexity of the experimental design, the possibility of type I errors cannot be excluded. Hence, the results need to be interpreted with caution [41]. All analyses were performed using the statistical software R 2.15.1 (R Development Core Team).

### Ethics statement

This study was approved by the Ethical Committee of the Agricultural Office of the Canton of Bern, Switzerland (experimentation permit BE 19/13 to AB). Bird catching and ringing were performed with permission of the Federal Agency for the Environment of the Canton of Bern, Switzerland (ringing permit 2992). During the measurement, eggs were carefully stored in small cotton-padded boxes and kept warm. The entrance of the nest was blocked to prevent females from entering and finding an empty nest. The same procedure was used during the cross-fostering of the nestlings. Nestlings were marked by selectively removing combinations of tuft feathers, which are lost naturally once the head feathers grow. Less than 5 μL (<1% of the body mass and <0.4% of the total blood volume [42]) of blood was taken from nestlings for sexing.

Our treatment did not increase the probability of nest desertion (Fisher’s exact test: *χ* ^2^ = 3.579, *P* = 0.167).

## Results

### Incubation rhythm and temperature

**Fig 2:**
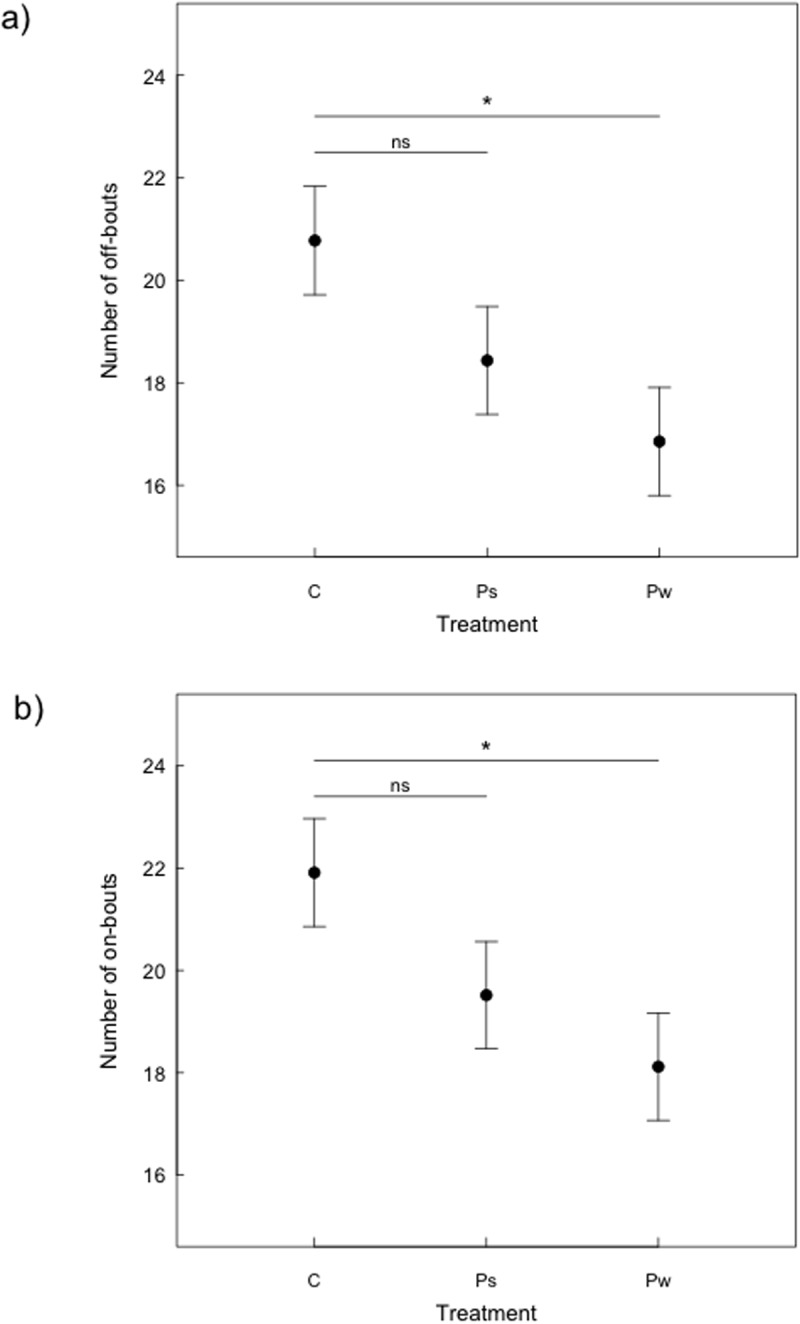
Average number of on-and off-bouts (mean ± SE) from a GLMM with Poisson error structure. (a) Females incubating in increased nest predation risk environments (Pw) had a lower number of off-bouts/active day compared to the control. Females under an increased post-fledging predator risk (Ps) had a tendency for a lower number of off-bouts compared to the control (*P* = 0.008). (b) Females incubating in increased nest predation risk environments (Pw) had a significantly lower number of on-bouts/active day compared to the control. Females under an increased post-fledging predator risk (Ps) had a slightly but non significantly lower number of on-bouts compared to the control (*P* = 0.032).

All details related to these analyses are provided in Table 1. The predator treatment had a significant effect on the number of times females left the nest during the active day (χ^2^_2_ = 9.665, *P* = 0.008; Fig. 2a): the number of off-bouts per day was highest for females in the control group (mean±SE: (20.8 ± 1.1)/day), slightly but not significantly lower in the Ps group (mean±SE: (18.4 ± 1.0)/ day), and significantly lower in the Pw group (mean±SE: (16.8 ± 1.0)/ day). Also the number of on-bouts was highest for females in the control group (mean±SE: (21.9 ± 1.1)/day), not significantly lower for females in the Ps group (mean±SE: (19.5 ± 1.0)/day) and significantly lower in the Pw group (mean±SE: (18.1 ± 1.1)/day) (χ^2^_2_ = 6.851, *P* = 0.032; Fig. 2b).

**Table 1:** ANOVA table of LMMs and GLMs for incubation behaviour.

Measurement	Variable	Standardized estimate	CI	df	χ^2^	*P*
Average number of off-bouts (family Poisson)	**Intercept**	**3.073**	**2.976/3.170**	—-	—-	—-
	**Average ambient temp**.	**0.064**	-**0.018/0.146**	**1**	**2.365**	**0.124**
	**Average daily precipit**.	**0.003**	-**0.056/0.063**	**1**	**0.012**	**0.914**
	**Laying-date**	**0.035**	-**0.046/0.115**	**1**	**0.710**	**0.399**
	**Clutch size**	-**0.005**	-**0.058/0.047**	**1**	**0.042**	**0.837**
	**Incubation day**	**0.019**	-**0.008/0.046**	**1**	**1.949**	**0.163**
	**Predator treatment**			**2**	**9.665**	**0.008**
	**Ps**	-**0.119**	-**0.253/0.015**			
	**Pw**	-**0.209**	-**0.341/-0.077**			
	Predator treatment X Incubation day			2	1.971	0.373
	Ps X Incubation day	0.015	-0.040/0.081			
	Pw X Incubation day	0.048	-0.021/0.117			
Average number of on-bouts (family Poisson)	**Intercept**	**3.087**	**2.982/3.191**	—-	—-	—-
	**Average ambient temp**.	**0.081**	-**0.008/0.169**	**1**	**3.213**	**0.073**
	**Average daily precipit**.	-**0.008**	-**0.073/0.056**	**1**	**0.063**	**0.801**
	**Laying-date**	**0.045**	-**0.042/0.132**	**1**	**1.036**	**0.309**
	**Clutch size**	**0.002**	-**0.055/0.059**	**1**	**0.003**	**0.953**
	**Incubation day**	**0.021**	-**0.005/0.047**	**1**	**2.439**	**0.118**
	**Predator treatment**			**2**	**6.851**	**0.032**
	**Ps**	-**0.116**	-**0.260/0.030**			
	**Pw**	-**0.190**	-**0.332/-0.047**			
	Predator treatment X Incubation day			2	1.002	0.606
	Ps X Incubation day	-0.005	-0.040/0.093			
	Pw X Incubation day	0.027	-0.068/0.059			
Measurement	Variable	Standardized estimate	CI	df	*F*	*P*
Duration of incubation	**Intercept**	**12.520**	**11.834/13.207**	—-	—-	—-
	**Average ambient temp**.	**0.170**	-**0.306/0.645**	**1, 48**	**0.515**	**0.476**
	**Average daily precipit**.	**0.132**	-**0.204/0.469**	**1, 48**	**0.623**	**0.434**
	**Laying-date**	**0.319**	-**0.135/0.773**	**1, 48**	**1.999**	**0.164**
	**Clutch size**	**0.311**	-**0.067/0.690**	**1, 48**	**2.733**	**0.105**
	**Predator treatment**			**2, 6**	**0.122**	**0.887**
	**Ps**	**0.077**	-**1.034/1.189**			
	**Pw**	**0.218**	-**0.898/1.334**			
Incubation constancy	**Intercept**	**35444.61**	**34001.5/36887.7**	—-	—-	—-
	**Average ambient temp**.	**898.21**	-**252.27/2048.69**	**1, 33**	**2.523**	**0.122**
	**Average daily precipit**.	-**32.26**	-**860.31/795.78**	**1, 33**	**0.006**	**0.937**
	**Laying-date**	**909.25**	-**240.24/2058.74**	**1, 33**	**2.590**	**0.117**
	**Clutch size**	**239.94**	-**466.69/946.58**	**1, 33**	**0.477**	**0.494**
	**Incubation day**	**642.88**	**351.67/934.08**	**1, 225**	**18.925**	**<0.001**
	**Predator treatment**			**2, 6**	**0.023**	**0.977**
	**Ps**	-**1.81**	-**2507.7/2504.1**			
	**Pw**	**175.47**	-**2272.7/2623.7**			
	Predator treatment X Incubation day			2, 223	0.185	0.831
	Ps X Incubation day	-6,45	-730.7/717.8			
	Pw X Incubation day	187.31	-553.1/927.8			
log (Average off-bout duration)	**Intercept**	**6.231**	**6.109/6.352**	—-	—-	—-
	**Average ambient temp**.	-**0.032**	-**0.141/0.077**	**1, 34**	**0.361**	**0.552**
	**Average daily precipit**.	-**0.016**	-**0.092/0.060**	**1, 34**	**0.186**	**0.669**
	**Laying-date**	-**0.065**	-**0.172/0.043**	**1, 34**	**1.500**	**0.229**
	**Clutch size**	-**0.060**	-**0.131/0.011**	**1, 34**	**2.963**	**0.094**
	**Incubation day**	-**0.074**	-**0.094/-0.054**	**1, 226**	**52.727**	**<0.001**
	**Predator treatment**			**2, 6**	**1.998**	**0.216**
	**Ps**	**0.132**	-**0.077/0.341**			
	**Pw**	**0.161**	-**0.043/0.364**			
	Predator treatment X Incubation day			2, 224	0.670	0.513
	Ps X Incubation day	0.002	-0.049/0.052			
	Pw X Incubation day	-0.024	-0.077/0.029			
log (Average on-bout duration)	**Intercept**	**6.396**	**6.190/6.602**	—-	—-	—-
	**Average ambient temp**.	-**0.086**	-**0.263/0.091**	**1, 34**	**0.980**	**0.329**
	**Average daily precipit**.	-**0.080**	-**0.210/0.049**	**1, 34**	**1.598**	**0.215**
	**Laying-date**	-**0.048**	-**0.223/0.127**	**1, 34**	**0.311**	**0.581**
	**Clutch size**	**0.025**	-**0.088/0.139**	**1, 34**	**0.204**	**0.654**
	**Incubation day**	-**0.066**	-**0.089/-0.044**	**1, 226**	**34.170**	**<0.001**
	**Predator treatment**			**2, 6**	**2.082**	**0.206**
	**Ps**	**0.268**	-**0.087/0.622**			
	**Pw**	**0.254**	-**0.093/0.601**			
	Predator treatment X Incubation day			2, 224	0.870	0.420
	Ps X Incubation day	-0.024	-0.075/0.027			
	Pw X Incubation day	-0.034	-0.086/0.018			
On-bout temperature	**Intercept**	**28.200**	**25.317/31.082**	—-	—-	—-
	**Average ambient temp**.	**2.001**	-**0.309/4.311**	**1, 31**	**3.122**	**0.087**
	**Average daily precipit**.	**0.080**	-**1.612/1.771**	**1, 31**	**0.009**	**0.924**
	**Laying-date**	**1.457**	-**0.816/3.731**	**1, 31**	**1.708**	**0.201**
	**Clutch size**	**1.012**	-**0.522/2.547**	**1, 31**	**1.811**	**0.188**
	**Incubation day**	-**0.853**	-**1.170/-0.536**	**1, 213**	**18.220**	**<0.001**
	**Incubation day** ^**2**^	**0.255**	**0.072/0.437**	**1, 213**	**7.579**	**0.006**
	**Predator treatment**			**2, 6**	**1.993**	**0.217**
	**Ps**	-**2.800**	-**7.607/2.007**			
	**Pw**	-**2.073**	-**6.880/2.733**			
	**Predator treatment X Incubation day**			**2,213**	**4.330**	**0.014**
	**Ps X Incubation day**	**0.583**	**0.188/0.979**			
	**Pw X Incubation day**	**0.431**	**0.017/0.846**			
Off-bout temperature	**Intercept**	**22.452**	**20.700/24.204**	—-	—-	—-
	**Average ambient temp**.	**1.162**	-**0.238/2.562**	**1, 31**	**2.865**	**0.100**
	**Average daily precipit**.	-**0.078**	-**1.103/0.947**	**1, 31**	**0.024**	**0.877**
	**Laying-date**	**0.891**	-**0.488/2.269**	**1, 31**	**1.737**	**0.197**
	**Clutch size**	**1.194**	**0.264/2.124**	**1, 31**	**6.859**	**0.013**
	**Incubation day**	-**0.133**	-**0.313/0.047**	**1, 215**	**5.079**	**0.025**
	**Incubation day** ^**2**^	**0.211**	**0.018/0.403**	**1, 215**	**4.653**	**0.032**
	**Predator treatment**			**2, 6**	**0.341**	**0.724**
	**Ps**	-**0.969**	-**3.881/1.942**			
	**Pw**	-**0.727**	-**3.638/2.184**			
	Predator treatment X Incubation day			2,213	1.114	0.330
	Ps X Incubation day	0.293	-0.125/0.711			
	Pw X Incubation day	0.280	-0.158/0.719			

Terms retained in the final model are highlighted in bold. Values for non-significant interactions represent values just before removal (significance level for interactions *P* < 0.05). The reference level for the coefficients is the control treatment. CI are 95% lower and upper confidence limits.

**Fig 3:**
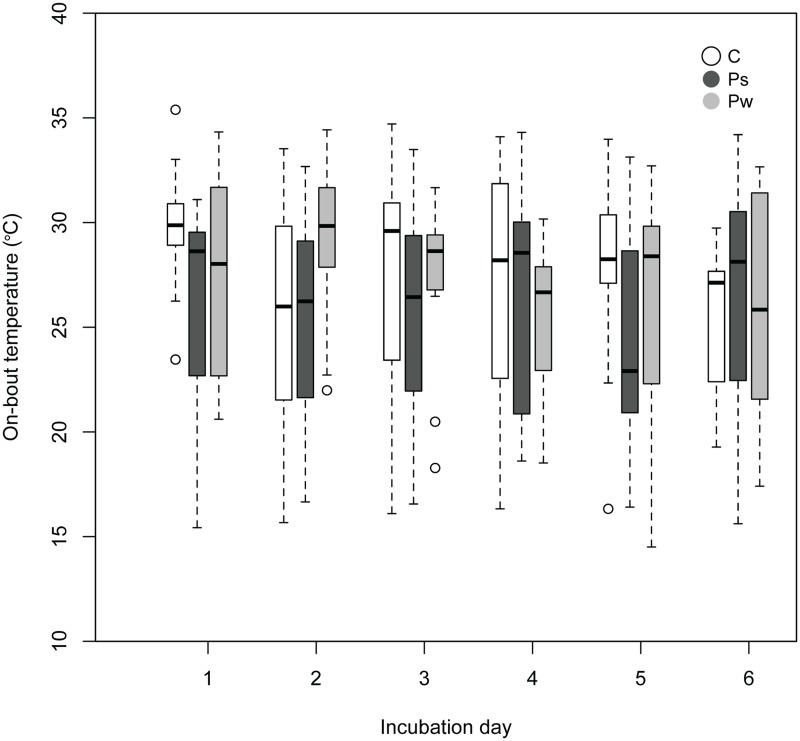
Box-plots of average on-bout temperature for the three treatment groups in the different incubation days. In both predator treatments temperature had a slower decline as incubation advanced compared to the control group (*P* = 0.014).

Predator treatment did not affect the total duration of incubation in the whole breeding period (*F*_2,6_ = 0.122, *P* = 0.887). Also incubation constancy (i.e. the amount of time the female spent incubating during the day) was not significantly affected by the predator treatment (*F*_2,6_ = 0.023, *P* = 0.977), or by the interaction between treatment and day of incubation (*F*_2,223_ = 0.185, *P* = 0.831). However, incubation constancy increased as incubation advanced (*F*_1,225_ = 18.925, *P*<0.001). None of the predator treatments, nor the interaction between treatment and day of incubation significantly influenced average off-bout duration (predator treatments: *F*_2,6_ = 1.998, *P* = 0.216; interaction predator treatments X day of incubation: *F*_2,224_ = 0.670, *P* = 0.513), but duration of time the female spent off the nest significantly decreased as the incubation progressed (*F*_1,226_ = 52.727, *P*<0.001). Average on-bout duration was also not significantly affected by the predator treatments or by the interaction between treatment and day of incubation (predator treatments: *F*_2,6_ = 2.082, *P* = 0.206; interaction predator treatments X day of incubation: *F*_2,224_ = 0.870, *P* = 0.420), but there was a significant decrease in average duration as incubation advanced (*F*_1,226_ = 34.170, *P*<0.001). On-bout temperature, i.e. average temperature of the on-bouts on each active day, was significantly affected by the interaction between day of incubation and predator treatment (*F*_2,213_ = 4.330, *P* = 0.014; Fig. 3), showing a slower decline in incubation temperature as incubation advanced in both predator treatments compared to control nests (Ps X day of incubation [linear]: 0.583, 95% CI = 0.188/0.979; Pw X day of incubation: 0.431, 95% CI = -0.017/0.846). On-bout egg temperature over the incubation period followed a quadratic function (polynomial incubation day [quadratic]: *F*_1,213_ = 7.579, *P* = 0.006). No significant interaction (*F*_2,213_ = 1.114, *P* = 0.330) or main effect of the treatment (*F*_2,6_ = 0.341, *P* = 0.724) was detected on off-bout temperature.

### Other incubation parameters

Incubation feeding (the number of times the male fed the female during incubation) was not significantly influenced by any of the predator treatments (Ps: 0.307, 95% CI = -1.217/1.831; *P* = 0.693; Pw: -0.265, 95% CI = -1.900/1.369, *P* = 0.750). However, when restricting the analyses to the nests in which males actually fed females at the nest, incubation feeding significantly increased in the Ps group compared to the control (0.955, 95% CI = 0.403/1.508, *P*<0.001), while no significant difference was detected for the Pw group (0.362, 95% CI = -0.232/0.955, *P* = 0.232).

### Nestling growth

**Fig 4:**
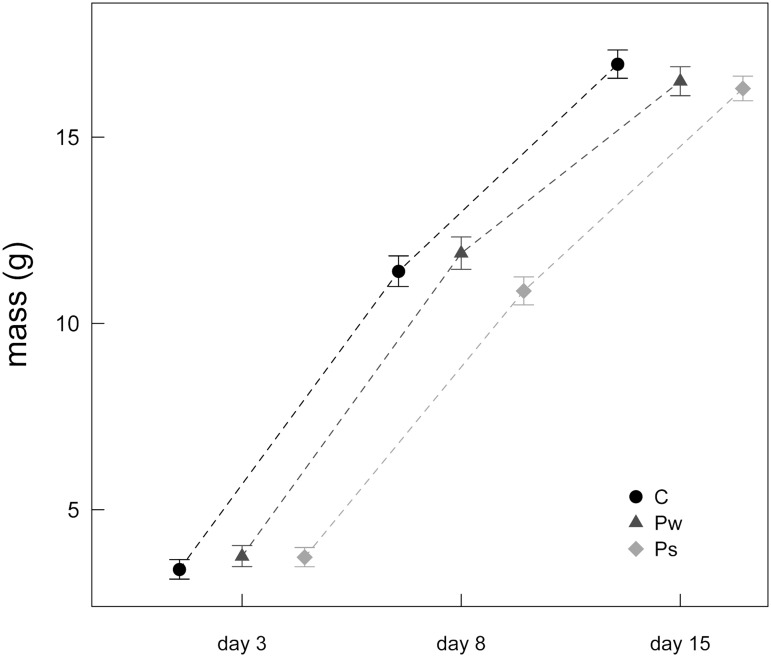
Nestling mass (mean±SE) on three measurement days. The shape of growth curves differed significantly according to the interaction between the treatments (*P*<0.001). Growth was slower from day 3 to day 8 in the Ps group and from day 8 to day 15 in the Pw group. C = mothers exposed to control treatment; Ps = mothers exposed to the sparrowhawk predator treatment; Pw = mothers exposed to the weasel predator treatment.

All the details related to these analyses are provided in Table 2. Brood size on day 3 did not differ significantly among the treatments (Ps: -0.078, 95% CI = -1.339/1.182; Pw: 0.192, 95% CI = -1.140/1.525; *F*_2,6_ = 0.186, *P* = 0.835). Nestling growth, measured by mass gain and tarsus growth, showed no correlation (*P*>0.05) with any of the incubation parameters (S1 Table). In the model of mass gain, the interaction among the maternal predator treatments and nestling age was significant (*F*_4,609_ = 13.604, *P*<0.001; Fig. 4). The analysis of mass gain in the different treatment groups between days 3–8 and between days 8–15 showed that both maternal predator treatments negatively affected nestling mass gain (slower and/or later growth), but this was significant only from day 3 to day 8 in the Ps group (Ps X age_8_: -0.175, 95% CI = -0.233/-0.117), and from day 8 to day 15 in the Pw group (Pw X age_15_: -0.078, 95% CI = -0.144/-0.011). We did not find any significant effect of the interaction between maternal predator treatments and nestling sex (*F*_2,270_ = 2.908, *P* = 0.056). No significant treatment-specific difference was found in the analyses of tarsus growth (all *P* values>0.07, Table 2). There was also no significant effect of the treatments on fledging age (*F*_2,6_ = 0.176, *P* = 0.843) and on fledging success (χ^2^_2_ = 1.077, *P* = 0.584).

**Table 2:** ANOVA table of LMMs for nestling and growth.

Measurement	Variable	Standardized estimate	CI	df	F	*P*
Mass	**Intercept**	**1.807**	**1.696/1.918**	—-	—-	—-
	**Brood size**	-**0.032**	-**0.065/0.001**	**1, 39**	**3.839**	**0.057**
	**Hatching date**	-**0.058**	-**0.093/-0.023**	**1, 39**	**11.055**	**0.002**
	**Sex**	**0.028**	-**0.009/0.064**	**1, 272**	**2.181**	**0.141**
	**Age**			**2, 609**	**5654.9**	**<0.001**
	**age 8**	**1.585**	**1.542/1.629**			
	** age 15**	**2.321**	**2.278/2.365**			
	**Predator treatment**			**2, 6**	**1.114**	**0.388**
	**Ps**	**0.090**	-**0.096/0.276**			
	** Pw**	**0.110**	-**0.085/0.304**			
	Predator treatment X sex			2, 270	2.908	0.056
	m X Ps	0.051	-0.420/0.522			
	m X Pw	-0.519	-1.033/-0.005			
	**Predator treatment X age**			**4, 609**	**13.604**	**<0.001**
	** age 8 X Ps**	-**0.175**	-**0.233/-0.117**			
	** age 15 X Ps**	-**0.181**	-**0.241/-0.121**			
	** age 8 X Pw**	-**0.046**	-**0.111/0.020**			
	** age 15 X Pw**	-**0.124**	-**0.191/-0.057**			
Tarsus	**Intercept**	**15.552**	**15.221/15.883**	—-	—-	—-
	**Brood size**	-0.028	-**0.189/0.133**	**1, 39**	**0.123**	**0.728**
	**Hatching date**	-**0.247**	-**0.427/-0.067**	**1, 39**	**7.735**	**0.008**
	**Sex**	**0.220**	**0.058/0.382**	**1, 272**	**7.177**	**0.008**
	**Age**			**1, 292**	**2458.8**	**<0.001**
	** age 15**	**3.296**	**3.165/3.426**			
	**Predator treatment**			**2, 6**	**0.357**	**0.714**
	** Ps**	-**0.045**	-**0.544/0.454**			
	** Pw**	**0.131**	-**0.424/0.686**			
	Predator treatment X sex			2, 270	2.697	0.069
	m X Ps	0.080	-0.298/0.458			
	m X Pw	-0.380	-0.793/0.033			
	Predator treatment X age			2, 290	2.322	0.100
	age 15 X Ps	0.163	-0.141/0.467			
	age 15 X Pw	-0.198	-0.535/0.138			

Terms retained in the final model are highlighted in bold. Values for non-significant interactions represent values just before removal (significance level for interactions *P* < 0.05). The reference level for the coefficients is a female nestling under control treatment. CI are 95% lower and upper confidence limits.

## Discussion

Studies on incubation behaviour under increased predation risk found variation in female strategies [43,44] when facing the trade-off between self-maintenance and investment in embryonic development during incubation [45,46]. This variation is reflected in behavioural and physiological responses (such as reduction of activity around the nest, lower parental care, hormone allocation in the eggs, etc.). Within species, this variation may be caused by different trade-offs in reproductive investment [4,20]. In this study, we assessed i) whether incubating females exposed to different predators that pose a threat either outside or inside the nest cavity would use different incubation behaviours to specifically cope with the two threats and ii) possible effects of predator-induced variation in incubation behaviour on offspring after hatching.

### Incubation

We predicted that on- and off-bouts, egg temperature and incubation constancy would change in response to the presence of either a nest predator or of a predator targeting adults during incubation and offspring after fledging. Among the measures related to incubation behaviour, only the number of on- and off-bouts and the on-bout temperature differed among predator treatments. Following our predictions, the number of on- and off-bouts under increased nest predation risk was lower compared to the control treatment, while a tendency only for a decreased number of on- and off-bouts was observed when predation risk was increased for adults outside the nest. This outcome is in line with previous studies where the frequency of movements close to the nest was reduced in the presence of a nest predator, possibly to minimize visual cues (e.g. [4,7,19,47]). The slightly but not significantly lower number of off-bouts in the group exposed to sparrowhawks may slightly lower the risk to be predated during one of the foraging trips [5]. These results might partly depend also on male incubation feeding, which can be increased to assist the female in the aim of maximizing the rate of embryonic development, minimizing the time parents are exposed to predators while remaining close to the nest. However, increased male incubation feeding (considering the nests in which males actually fed females during the recording) was detected in our study only for the nests exposed to a post-fledging predator. A potential effect of nest predation risk on male incubation feeding cannot however be excluded, as the recordings were made at the beginning of the incubation period only inside the nest boxes. Male great tits may choose to feed their mate outside the nest or to lead them to good foraging patches [16].

A clearer understanding of female investment in current reproduction or on self-maintenance would require a link between frequency of movements to and from the nest (on- and off-bouts) and other incubation parameters (i.e. on- and off-bout duration, incubation temperature and constancy). If females would prioritize investment in the current clutch, fewer off-bouts should be reflected in an increased time on the nest, achieved at the expenses of foraging. Consequently, resource-depleted females may in turn reduce steady-state incubation temperatures to achieve increased incubation constancy [34]. According to the previous hypothesis, during the on-bouts incubation temperature decreased as incubation advanced following a quadratic relationship, while incubation constancy increased. However, only on-bout temperature showed a slower decline in time under both predator treatments, but predation risk had no effect on off-bout temperature, on- and off- bout duration, or on incubation constancy making the interpretation of this result rather difficult. Perhaps timing of exposure to the predators might not have been long enough to trigger stronger responses. Besides, although we controlled for rainfall and ambient temperatures in all our models, unusually low temperatures and high precipitation during May 2013 could have affected these results, by influencing bird reproductive success and parental investment, and therefore potentially mask some of the treatment effects (average temperatures of 1.5 to 3.5°C below the average temperatures recorded in 1981–2010, and by precipitations of 130 to 200% higher compared to the average (http://www.meteosuisse.admin.ch)).

### Nestling growth

As increased predation risk (inside and outside the nest) affected some aspect of female incubation behaviour (i.e. off-bout frequency and on-bout temperature), we explored possible effects of the incubation rhythm on nestling growth.

The decreased number of off-bouts in the presence of a nest predator and the slower decline of incubation temperatures in the presence of both types of predators may intuitively suggest enhanced development of offspring born from mothers exposed to predation risk since eggs were less exposed to frequent cooling and temperature fluctuations. Indeed, it has been demonstrated that variation in incubation investment with high temperature fluctuations may be directly harmful for developing young [22,48]. However, nestlings in both maternal treatments seems to show a different scenario, since nestling mass gain was slower when mothers were exposed to both nest predators and post-fledging predators. Surprisingly, nestlings whose mothers were exposed to a high nest predation risk showed reduced mass gain in later phases of growth, while under a post-fledging predation risk mass gain was negatively affected only in earlier phases. Nevertheless, direction of the estimates showed a general negative trend for growth rates under predator treatment, independently of nestling age. Independently of incubation treatment, slight changes in incubation temperature may influence stress hormone profiles of offspring, which may also influence embryonic developmental conditions and juvenile fitness [49,50]. Stressful conditions during incubation might also affect the nestling rearing phase. In the study of Cichon [51], experimentally increased costs of incubation (i.e. clutch size manipulation) in collared flycatchers showed offspring in poorer body condition after hatching. Even though we found no evidence for an effect of the predator treatments on either fledging probability or fledging age, we cannot exclude possible carry-over effects on later life-history stages, since nestling size at the time of fledging is often associated with different fitness-related traits, such as future survival and fecundity [20,52,53].

In conclusion, experimentally increased predation risk during incubation affected some aspects of female incubation behaviour (i.e. on- and off-bout frequency and on-bout temperature) as a function of the predator type. Even though the assessment of the direction and amplitude of female reproductive investment remains unclear and effect sizes seem to be rather small, both predator treatments induced modifications in incubation behavior, which appear to negatively affect the nestling condition. Predator-specific effects on incubation behaviour deserve further investigation, and the present study can be useful in guiding future work on predator-specific effects on incubation behaviour.

## Supporting Information

S1 Table Correlation among incubation parameters and nestling morphological traits. (DOCX)
