# Supplementary material for: Predator-Specific Effects on Incubation Behaviour and Offspring Growth in Great Tits
Source: PLoS One. 2015 Apr 1;10(4):e0121088. doi: 10.1371/journal.pone.0121088 (PMC4382303; doi:10.1371/journal.pone.0121088)
Supplement: S1 Table — (DOCX) [file pone.0121088.s001.docx]

**S1 Table.** Correlation among incubation parameters and nestling morphological traits.

| Incubation parameter | Nestling mass gain | | Nestling tarsus growth | |
| --- | --- | --- | --- | --- |
|  | *r* | *P* | *r* | *P* |
| On-bout temperature | 0.024 | 0.44 | 0.017 | 0.669 |
| Off-bout temperature | 0.039 | 0.206 | 0.028 | 0.482 |
| Incub. constancy | 0.081 | 0.009 | 0.058 | 0.140 |
| N° of off-bouts | <0.001 | 0.999 | <0.001 | 0.995 |
| Off-bout duration | -0.051 | 0.099 | -0.037 | 0.344 |
